# Experimental validation of a low-cost maximum power point tracking technique based on artificial neural network for photovoltaic systems

**DOI:** 10.1038/s41598-024-67306-0

**Published:** 2024-08-07

**Authors:** Ahmed Fathy Abouzeid, Hadeer Eleraky, Ahmed Kalas, Rawya Rizk, Mohamed Mohamed Elsakka, Ahmed Refaat

**Affiliations:** 1 Electrical Engineering Department, Port Said University, Port Said, 42526 Egypt; 2 Mechanical Power Engineering Department, Port Said University, Port Said, 42526 Egypt

**Keywords:** Photovoltaic systems (PV), Maximum power point tracking (MPPT), Perturb & observe (P&O), Incremental conductance (IC), Artificial neural networks (ANNs), Electrical and electronic engineering, Photovoltaics

## Abstract

Maximum power point tracking (MPPT) is a technique involved in photovoltaic (PV) systems for optimizing the output power of solar panels. Traditional solutions like perturb and observe (P&O) and Incremental Conductance (IC) are commonly utilized to follow the MPP under various environmental circumstances. However, these algorithms suffer from slow tracking speed and low dynamics under fast-changing environment conditions. To cope with these demerits, a data-driven artificial neural network (ANN) algorithm for MPPT is proposed in this paper. By leveraging the learning capabilities of the ANN, the PV operating point can be adapted to dynamic changes in solar irradiation and temperature. Consequently, it offers promising solutions for MPPT in fast-changing environments as well as overcoming the limitations of traditional MPPT techniques. In this paper, simulations verification and experimental validation of a proposed data-driven ANN-MPPT technique are presented. Additionally, the proposed technique is analyzed and compared to traditional MPPT methods. The numerical and experimental findings indicate that, of the examined MPPT methods, the proposed ANN-MPPT approach achieves the highest MPPT efficiency at 98.16% and the shortest tracking time of 1.3 s.

## Introduction

The necessity to seek out clean and renewable energy sources has been significantly expanded due to the development of the current industrial civilization, population expansion, and environmental concerns. Photovoltaic (PV) solar energy is recognized to be one of the more recent categories of fastest-growing renewable energy sources because of its numerous benefits like low operating costs, lack of noise or mechanical moving parts, absence of emission of CO_2_ or other pollutants, and simplicity of use with both stand-alone and grid-connected systems^1^. However, high start-up costs, poor efficiency of conversion (around 12–25%), nonlinearity behavior, and weather dependency make PV systems hard to employ in large areas^2^. Therefore, PV system efficiency must be improved by operating at the highest possible power level to overcome these limitations for widespread.

Different maximum power point tracking (MPPT) methods have been presented in the literature. The presented techniques vary in terms of sensor requirements, hardware installation, dependence on PV module characteristics, dynamic reaction under unexpected changes in the environment, and tracking precision^3–13^. The perturb and observe (P&O) method is widely utilized for determining the maximum power of photovoltaic (PV) systems PV systems because of its simplicity^14,15^. This technique involves continuously adjusting the output voltage of the PV panel and subsequently comparing the output power with its previous value throughout the perturbation cycle. The control system modifies the operating point in accordance with changes in the operating voltage and increase in power experienced by the PV module^16^. Alternatively, the Incremental Conductance (IC) method, which is a model-based technique, can be found as a good competitor to P&O^17–19^. Utilizing voltage and current signals along with their derivatives, the IC method computes the derivative of the solar panel’s output power. The MPP is attained when the instantaneous conductance equals the incremental conductance. P&O and IC techniques are part of conventional ways of determining the MPPT, which take advantage of a PV panel’s (P–V) properties^20–22^.

Advanced MPPT based on Metaheuristic algorithms can be found in^23–25^. Metaheuristic algorithms are particularly made to address complicated issues involving several variables and find the best results. These algorithms employ several candidates to locate a possible solution during every iteration. Precision or speed may be improved by using several agents. However, it can also result in decreased energy tracking. Particle Swarm Optimization (PSO) is an efficient optimization approach for harvesting the most power possible from photovoltaic modules across diverse environmental conditions. PSO can effectively explore the field of search and identify optimum solutions by emulating the social behavior of particles within a swarm^26,27^. Alternative bio-inspired optimization algorithms such as Cuckoo Search (CS) optimizer and Gray Wolf (GW) optimizer can be involved in searching for the MPP of the solar panels. GW is influenced by gray wolves’ social structure and hunting methods while the CS method effectively traverses the search space and converges to near-optimal solutions by imitating the nesting and parasitism behavior of cuckoo birds^28–31^.

In recent times, there have been significant progressions in applying Artificial Intelligence (AI)-based techniques for MPPT due to their potential to significantly improve the reliability and efficiency of MPPT in PV systems. Machine learning algorithms, especially neural networks, have shown promising solutions to predict the optimal operating point for PV panels^32^. These algorithms utilize the capabilities of neural networks to learn and adapt to changing conditions, making them suitable for tracking the MPP. In^33^, the authors employ a multiple linear regression (MLR) model to forecast the PV efficiency based on the weather parameters. MLR is a conventional statistical modeling approach that assumes a linear connection between the predictors and the target variable. A feedforward ANN with a backpropagation algorithm is used to forecast efficiency. The results indicate that the ANN model surpasses the MLR model in terms of predictive accuracy, demonstrating lower mean root mean square error (RMSE), absolute error (MAE), and better R-squared values. This suggests superior performance in capturing the non-linear associations between weather parameters and efficiency. One limitation is that the analysis was conducted on data from only one PV power plant situated in a specific region. Therefore, the generalizability of the findings to other locations or different types of solar power plants may be limited. In^34^, the authors introduce an MPPT technique based on ANNs and compare its performance with traditional MPPT algorithms under various conditions, such as changing solar irradiance and temperature. The findings indicate the ANN-based technique surpasses traditional techniques in terms of both accuracy and efficiency. The limitation of the proposed technique in^34^ is evaluated through simulations without experimental validation to assess its performance in real conditions. Moreover, the cost analysis focuses on hardware complexity reduction without a detailed comparison with other ANN-based MPPT techniques, limiting the understanding of its uniqueness and novelty. An ANN-based MPPT technique under varying climatic conditions with a CUK converter topology is proposed in^35^. The presented topology demonstrates superior performance compared to traditional MPPT techniques, exhibiting enhanced accuracy, efficiency, and robustness. However, the presented system performance is evaluated through simulations only without discussing the potential challenges or limitations that may arise during the implementation of the suggested system in real PV systems. In^36^, ANN-based MPPT method is presented to follow the MPP even under rapidly variable shading conditions. The presented method is trained with a backpropagation algorithm to estimate the MPP voltage and current based on input variables like solar radiation and temperature considering various shading scenarios. The authors compare the performance of the presented method with two conventional MPPT techniques: P&O and IC. The primary merits of the presented method over conventional techniques include higher tracking accuracy and faster response time, especially when dealing with rapidly changing shading patterns. However, the main drawbacks of the presented method include the absence of experimental validation and its dependency on precise measurements of irradiance and temperature. In^37^, a comparative study is conducted on the performance of various ANN training methods for MPPT in photovoltaic systems. Six different algorithms, the Levenberg-Marquardt (LM), Broyden-Fletcher-Goldfarb-Shanno (BFGS), gradient descent momentum (GDM), Bayesian regularization (BR), scaled conjugate gradient (SCG), and resilient backpropagation (RP), are evaluated by simulations. It is found that the BFGS and LM methods have the highest performance in terms of maximum energy harvesting in PV systems. The main limitation of this study is the lack of experimental investigation and validation which is being covered in this paper.

In this paper, a novel MPPT technique based on ANN for PV systems is proposed. The proposed technique shows fast and accurate MPPT capability with low sensor requirements. The key contributions of this study can be outlined as follows:

1. A novel MPPT technique based on the data-driven ANN algorithm is proposed.
2. MATLAB simulations are conducted to evaluate the efficacy of the proposed ANN technique compared to traditional MPPT methods like P&O and IC in the presence of rapid changes in solar radiation and temperature.
3. The suggested ANN-MPPT technique is validated experimentally by using a commercial low-cost microcontroller and minimum sensor requirements.

The effectiveness of the proposed method surpasses that of both other tested techniques, exhibiting superior tracking capability with low power oscillations. Furthermore, the proposed approach achieves the lowest tracking time, significantly outperforming the IC and P&O methods. The tracking time of the ANN-MPPT is notably shorter compared to IC and P&O. The structure of the article is summarized as follows: Section “System description” Inprovides the PV system description under investigation; Section “Overview of existing MPPT techniques” includes an overview of the existing traditional MPPT methods; Section “Proposed data-driven MPPT technique using artificial neural network” includes the suggested ANN-MPPT method; Section “Results and discussion” includes findings and discussion; conclusions are finally discussed in section “Conclusion”.

## System description

**Figure 1:**
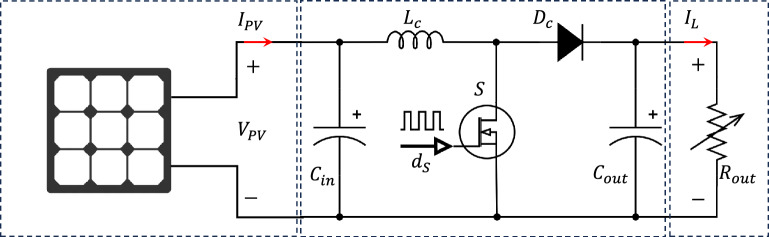
A schematic of a DC load connected to a PV panel via a controlled DC–DC boost converter.

S The schematic representation of a PV battery charging system is shown in Fig. 1. The system consists of a PV panel, a DC–DC step-up converter equipped with an MPPT controller, and a resistive load representing the battery’s internal resistance. The main elements of the system will be discussed in the following subsections.

### PV (solar) panel

The accuracy of solar energy source modeling (cells, modules, or arrays) is crucial for PV systems, particularly when analyzing the characteristics, forecasting the generation of electricity, and harvesting the maximum amount of power. The non-linear behavior between the output voltage and current presents a modeling challenge for PV modules. To replicate the actual operation of the PV devices under various environmental circumstances, the modeling of PV generators comprises an estimate of the I–V and P–V characteristic curves^38,39^.

**Figure 2:**
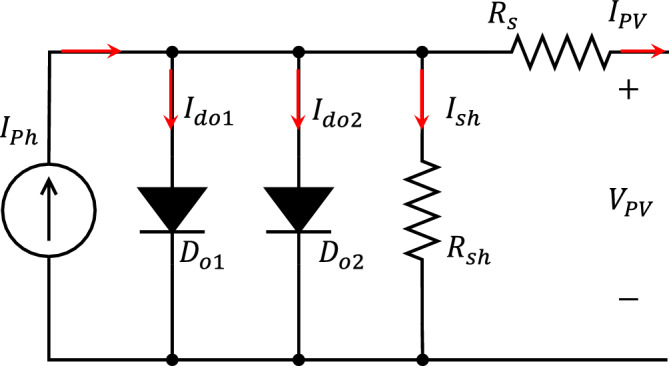
Double exponential PV cell equivalent circuit model.

The I–V characteristics of a realistic PV cell can be accurately represented by using the double exponential model depicted in Fig. 2 and expressed from (1) to (4). Where the voltage drop owing to the current passage via the semiconductor material is represented by the series resistance $R_s$. The leakage current to the ground at the cell limits is represented by the shunt resistance $R_{sh}$. The two diodes are used to replicate the non-linear characteristics of the solar cell, representing the impact of recombination within the depletion region. This recombination introduces a non-resistive current path running parallel to the intrinsic photovoltaic cell. It is crucial to emphasize that the ideality factors and saturation currents of the two diodes are distinct, leading to additional parameters to be estimated and increasing the complexity of the model^40^.

1 $$\begin{aligned} I_{PV}&=I_{ph}- I_{do1} - I_{do2} - I_{sh} \end{aligned}$$

2 $$\begin{aligned} I_{do1}&=I_{01}\left( \exp ^{\frac{q(V_{PV}+I_{PV} R_s)}{(m_1 k T_c )}}-1\right) \end{aligned}$$

3 $$\begin{aligned} I_{do2}&=I_{02}\left( \exp ^{\frac{q(V_{PV}+I_{PV} R_s)}{(m_2 k T_c )}}-1\right) \end{aligned}$$

4 $$\begin{aligned} I_{sh}&= \frac{V_{PV}+I_{PV} R_s}{R_{sh}} \end{aligned}$$

where $I_{PV}$: Output cell current, $V_{PV}$: Output cell voltage, $I_{ph}$: Photo-generated current, $I_{do1}$: The saturation current due to diffusion, $I_{do2}$: The saturation current due to recombination, $I_{sh}$: The shunt leakage current to ground.

**Figure 3:**
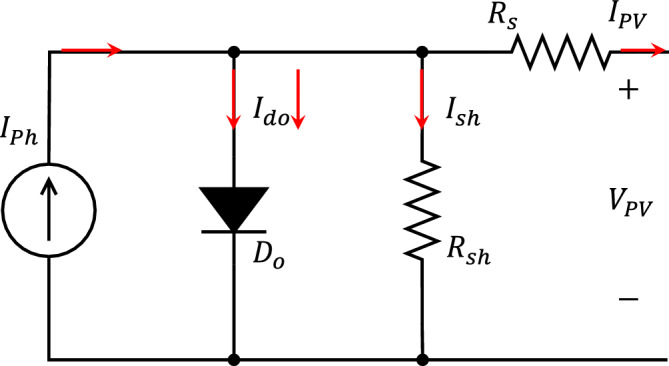
Single exponential PV cell equivalent circuit model.

In this work, the single exponential model is utilized to model the PV panels as shown in Fig. 3. This model strikes a good balance between model complexity and accuracy. Equation (5) describes the characteristic equation of a PV cell within the framework of this model^41,42^.

5 $$\begin{aligned} I_{PV}&=I_{ph}-I_{ph}\left( \exp ^{\frac{q(V_{PV}+I_{PV} R_s)}{(m k T_c )}}-1\right) - \left( \frac{V_{PV}+I_{PV} R_s}{R_{ah}}\right) \end{aligned}$$

For PV panel modelling, the parameters used are identical to those used in the PV cell, but the voltage is divided by the number of series-connected cells ($n_s$), the equation will be^43^:

6 $$\begin{aligned} I_{PV}&=I_{ph}-I_{ph}\left( \exp ^{\frac{(V_{PV}+I_{PV} R_s)}{(m V_t )}}-1\right) - \left( \frac{V_{PV}+I_{PV} R_s}{R_{ah}}\right) \end{aligned}$$

where $V_t=\frac{n_s k T_c}{q}$ is the panel thermal voltage.

### Boost converter

Boost converters are a crucial part of contemporary solar energy systems as they provide voltage step-up, allowing for long-distance power transmission from far away solar panels to load centers. Also, they make effective battery charging by matching the output voltage of the PV panels to the battery voltage including the maximum power point feature^44^. Boost converter is one of the common switched-mode DC–DC converter topologies employed to increase the solar module voltage to meet the load requirements. Additionally, maximize the power extraction from the PV panels by adjusting the converter duty cycle^43^. The boost converter, illustrated in Fig. 1, is used where energy is stored in the inductor when the semiconductor switch is on, and then the energy is released to the output when the switch is off. The capacitor produces a smooth DC output by filtering out any voltage ripple. The diode blocks the flow of reverse current from input to output. The switching operation using a power semiconductor switch (usually a MOSFET) that quickly turns on and off generates an output voltage depending on the switch duty cycle as in (7)^45^.

7 $$\begin{aligned} V_{in}&= V_{out} \left( 1-D\right) \end{aligned}$$

The main boost converter elements, i.e. the inductor and the capacitor, should be carefully designed to achieve fast dynamics without affecting the steady-state ripples. In this paper, the boost converter is designed to operate in the Continuous Conduction Mode (CCM) in order to reduce the current stress on the semiconductor switch. Using (8), the inductance of the boost converter, $L_{c}$, is obtained where $V_{out}$ is the load voltage, *D* denotes the duty cycle of the converter, $\Delta i_L$ denotes to the inductor ripple current, and $f_{sw}$ refers the switching frequency.

8 $$\begin{aligned} L_{c}&= \frac{V_{out} D}{\Delta i_L f_{sw}} \end{aligned}$$

The peak-to-peak capacitor ripple voltage is derived as in (9) where $C_{out}$ denotes the output capacitor.

9 $$\begin{aligned} V_{out}&= \frac{\Delta i_L}{8 f_{sw} C_{out}} \end{aligned}$$

The output capacitor voltage ripple factor is calculated as in (10) where $R_{out}$ refers to the load resistance.

10 $$\begin{aligned} \gamma _{V_{out}}&= \frac{D}{8 R_{out} C_{out} f_{sw}} \end{aligned}$$

For a proper operation of the boost converter, the voltage ripple factor should be maintained at low levels (recommended within 1%) and the current ripple is relatively larger than the voltage ripples. In this paper, a 15% of $\Delta i_L$ is chosen^46^.

### Optimal load selection

The output load of a PV system refers to the electrical load that the system produces. The output load is crucial because it establishes the solar panels’ power point at which they are producing their maximum power. The output load needs to be precisely adjusted to meet the solar panel’s (mpp) to determine the MPPT. Suppose an ideal system where the input power is equivalent to the output power, as depicted in (11).

11 $$\begin{aligned} \frac{V_{mpp}^2}{R_{mpp}}&= \frac{V_{out}^2}{R_{out}} = \frac{\left( \frac{V_{mpp}}{1-D}\right) ^2}{R_{out}} \end{aligned}$$

where $V_{mpp}$, $R_{mpp}$: are the voltage and load resistance at mpp respectively; $V_{out}$, $R_{out}$; are the output voltage and resistance respectively; and *D* is the duty cycle.

With various *D* and $R_{mpp}$, the range of $R_{out}$ is computed using (12) where the optimal selection of the load resistance is given in Table 1.

**Table 1:** Optimal output resistance selection for MPPT in PV systems.

Output resistance, $R_L$ [$\Omega$]	Irradiance, G [W/m^2^]	
	Maximum	Minimum
Maximum	$\frac{R_{{mpp}_{(min)}}}{(1-D_{max})^2}$	$\frac{R_{{mpp}_{(max)}}}{(1-D_{max})^2}$
Minimum	$\frac{R_{{mpp}_{(max)}}}{(1-D_{min})^2}$	$\frac{R_{{mpp}_{(min)}}}{(1-D_{min})^2}$
Optimum	$\frac{R_{{mpp}_{(max)}}}{(1-D_{min})^2} \le R_{out}\le \frac{R_{{mpp}_{(min)}}}{(1-D_{max})^2}$	

Thus, the duty cycle can be calculated from (12) knowing the load resistance.

12 $$\begin{aligned} D&= 1 - \sqrt{\frac{R_{mpp}}{R_{out}}} \end{aligned}$$

The optimal load is required as an indicator to operate the PV system at MPP because, while the MPPT algorithm adjusts the operating point to achieve maximum power output, an optimal load ensures the system consistently operates at or near this point, enhancing the overall efficiency and performance of the algorithm. When $R_{out}$ is outside of the ideal range (optimal), either drops below the minimum limit ($D_{min}$) or rises over the maximum limit ($D_{max}$). Nevertheless, $R_{out}$ optimal cannot be produced when the duty cycle range is excessively small^34,44^.

## Overview of existing MPPT techniques

### Perturb and observe (P&O)

**Figure 4:**
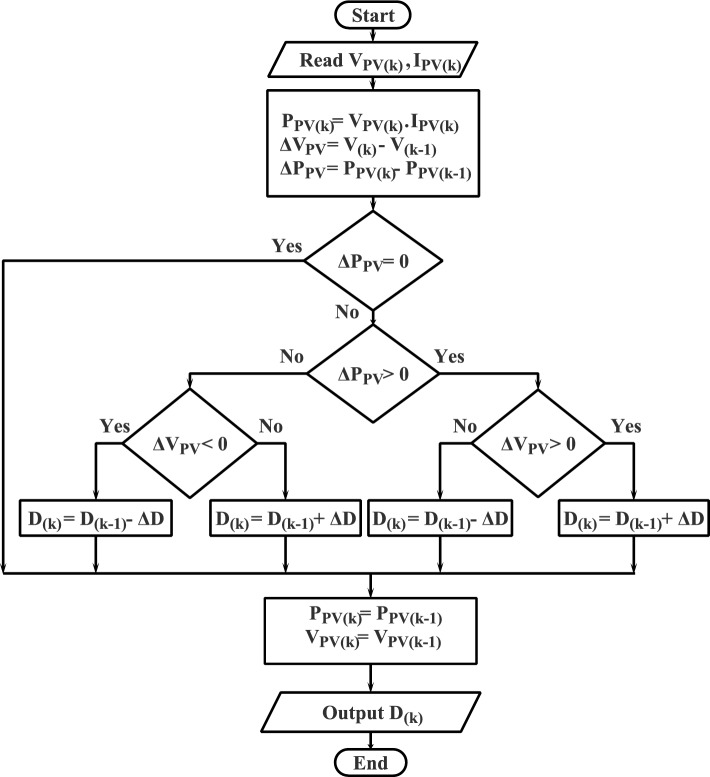
Flowchart of P&O MPPT technique.

The P&O is considered the most common MPPT techniques for PV systems due to its simplicity and lower computational requirements^44^. Basically, the P&O method operates by continually altering the PV system’s operating point while tracking any changes in power output. First, the PV system operates at a specific voltage or current level. Then, the operating point is slightly perturbed, and the change in the power output is being measured. Finally, the method decides whether to raise or lower the operating point for the following iteration based on the previous perturbation (see Fig. 4). Generally, the P&O algorithm’s objective is to identify the operating point that corresponds to the PV system’s maximum power output. To do this, the operating point is modified repeatedly until a local maximum is attained. Once a local maximum is detected, the algorithm reduces the perturbation step size to ensure that it operates around this maximum point without overshooting it^47,48^. The propensity to oscillate around the MPP, especially in situations with quickly varying environmental factors or partial shade, is the key downside of the P&O method. Consequently, a reduction in energy production and inefficient operation may result from these oscillations. Numerous improvements and adjustments have been proposed as a means of addressing this problem, including adaptive step size management, variable step size algorithms, and hybrid systems that combine P&O with other MPPT techniques^19–21^.

### Incremental conductance (IC)

**Figure 5:**
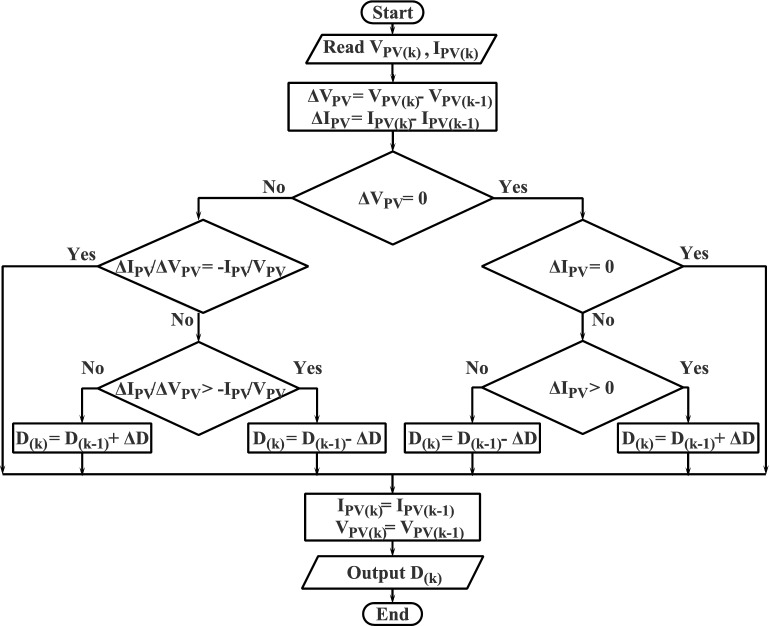
Flowchart of Incremental Conductance (IC) MPPT technique.

The strategy of the IC algorithm is found to be one of the most used MPPT approaches for PV systems due to its efficiency and simplicity^49^. The key objective of IC algorithm is to continually modify the operating point of the PV system to follow the MPP on the current-voltage (I–V) curve. By monitoring and comparing small changes in conductance, the MPP is determined, regardless of whether the operational point is approaching or moving away from the MPP. The derivative of power to voltage, or dP/dV, is what is referred to as conductance. Based on two variables (voltage and current), the IC algorithm continuously modifies the PV system’s voltage (see Fig. 5): a) Evaluation of Incremental Conductance: The method compares the instantaneous conductance (I/V) with the incremental conductance (dI/dV). The operating point is moving away from the MPP if I/V is smaller than dI/dV, the algorithm modifies the voltage in a way that moves it in the direction of the MPP. In contrast, if I/V is bigger than dI/dV. In this situation, the operating point is moving towards the MPP, and no modification is needed. b) Evaluation of Power: The algorithm additionally assesses the instantaneous power (P) in relation to its previous value (P_prev_). When P_prev_ exceeds P, the operating point is passed the MPP and the algorithm adapts the voltage toward the MPP. The IC method can successfully follow the MPP by continually measuring and modifying the voltage based on these two criteria. It guarantees that the photovoltaic system operates at its maximum output power across diverse load conditions and promptly responds to alterations in external conditions. Compared to the P&O method, the IC method has several benefits. Firstly, it is appropriate for a variety of PV installations as it doesn’t require any knowledge of the PV system specifications or features. The MPP can be precisely tracked even under quickly changing environmental circumstances since it is durable and dependable. Finally, it shows stable dynamic behavior, enabling effective power extraction from the solar panels^50^.

## Proposed data-driven MPPT technique using artificial neural network

### Overview

**Figure 6:**
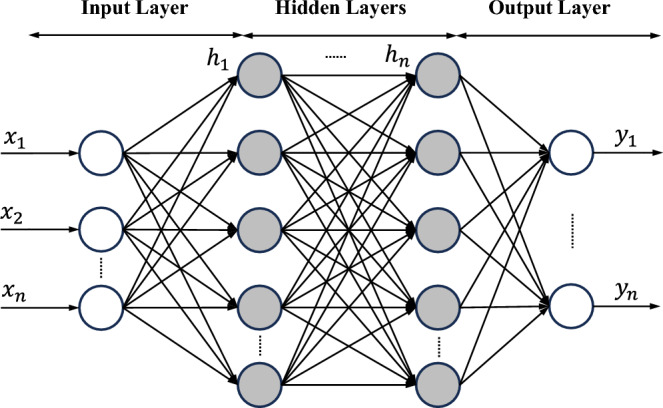
Schematic representation of the artificial neural network (ANN).

Data-driven methods have introduced alternative numerical analysis approaches. Traditional numerical analysis methods require solving a set of equations to represent the physical system. In the data-driven approaches, the system finds the minimum of the loss function by computing the possible solutions. artificial neural network (ANN) is one of the common approaches of data-driven techniques. The ANN is a computer model that is inspired by how natural neural networks of the human brain are organized and how to carry out their various functions. The ANN’s basic building blocks are linked nodes, often known as artificial neurons or just neurons, that are arranged into layers. These layers oversee handling the processing and transformation of incoming data into an output (see Fig. 6)^51^. The input layer, hidden layer(s), and output layer are the three primary elements of an ANN as follows:

1. **Input layer** The initial layer of a neural network acts as a conduit between the network and the outside world. It processes input data, which may be any kind of information that can be represented numerically, including text, photos, and numerical values. Each input layer neuron stands for a characteristic of the incoming data.
2. **Hidden layer** The hidden layer(s) are the intermediate level(s) between the input and output layers. They are essential for identifying intricate linkages and patterns in the data. Since these levels are not easily visible from outside the network, they are referred to as “hidden” layers. Each neuron in a hidden layer gets input from every other neuron in the layer below it (the input layer or another hidden layer), and it sends its output to every neuron in the layer above. Hidden layers in ANNs enable the learning of complicated representations and the application of non-linear modifications to the input data. To approximate complicated functions or mappings between inputs and outputs, ANNs can learn to modify the weights associated with each link between neurons. Depending on the application, the total number of hidden layers as well as the number of neurons inside each hidden layer might change. Networks having numerous hidden layers are known as deep neural networks, and they have achieved outstanding results in several fields. However, the complexity, computational effort, and processing time are the main demerits of increasing the layers and neurons number.
3. **Output layer** The output layer generates the desired result or prediction using the data from the processed input layer. The number of neurons in the output layer is determined by the nature of the problem being addressed^52,53^.

Feedforward neural networks (FNNs) provide promising solutions for MPPT in PV systems owing to their ability to address both linear and nonlinear problems [41]. FNNs may optimize the MPP by learning the relationship between the PV output power and the related MPP. This enhances the system’s efficiency as well as the generation of electricity. FNNs exhibit unidirectional information flow, moving solely from the input to the output. It is made up of several layers of connecting points (neurons). Each neuron takes its input from the previous layer, which performs a mathematical operation, and then transmits the changed values to the subsequent layer until it reaches the output layer. The FNN architecture aims to transform input data to output data without the need for any feedback connections or loops. This shows that the output of each neuron in a particular layer has no effect on neurons in previous levels. The FNN may be trained using previous data on different environmental variables like temperature, irradiance, and voltage/current measurements in the context of MPPT tracking for PV systems. Based on these inputs, the network can then predict the ideal operating point for maximum power extraction. It can adapt to changing situations and increase its accuracy over time by continually updating the input data and retraining the network. In MPPT control, an FNN is trained using a dataset of PV array output power and matching MPP values. During the training process, Mean Squared Error (MSE) or Mean Absolute Error (MAE) functions can be employed to minimize the difference between the forecasted MPP and the actual MPP. Upon training, the network can accurately forecast the MPP, enabling swift and precise Maximum MPPT control^54,55^. To reduce the mismatching between desired outputs and actual outputs, the weights and biases of the neural network’s linked nodes or neurons are adjusted during the training process where the parameters of the network are continuously updated using a feedback method which is called backpropagation. Backpropagation is a learning method that is involved in the training process of feedforward neural networks to correct network errors by adapting the weights and biases associated with each neuron. Then, the errors are propagated backward via the network, which is referred to as “backpropagation”. This process is repeated until the network errors are minimized and the network can reliably predict the expected results for a given set of inputs^51,55^.

### Methodology of the proposed technique

MPPT is one of the complicated topics in PV systems due to the nonlinearity and unpredictability of PV characteristics. Machine learning, specifically neural networks, provides a promising solution to cope with the demerits of traditional MPPT techniques^56,57^. The ANN-based MPPT algorithm utilizes the ability of neural networks to learn from data and make predictions or decisions without the need for manual programming. An ANN can understand the intricate correlations between these factors and correctly forecast the MPP by being trained using a dataset that includes inputs (such as sunlight irradiance, and temperature), and related outputs (such as power output). The ANN may be used in MPPT applications after the training process. The ANN technique generates an output signal that modifies the operating point of the PV panel for tracking the MPP using the measured input data^58^.

In this paper, two ANN-based MPPT techniques are introduced. The first ANN-based MPPT technique is the traditional one, where solar radiation (*G*) and module temperature (*T*) are the input variables to the ANN. The second ANN-based MPPT technique is the proposed one, where the PV module current ($I_{in}$) and module temperature (*T*) are the input variables to the ANN.

**Figure 7:**
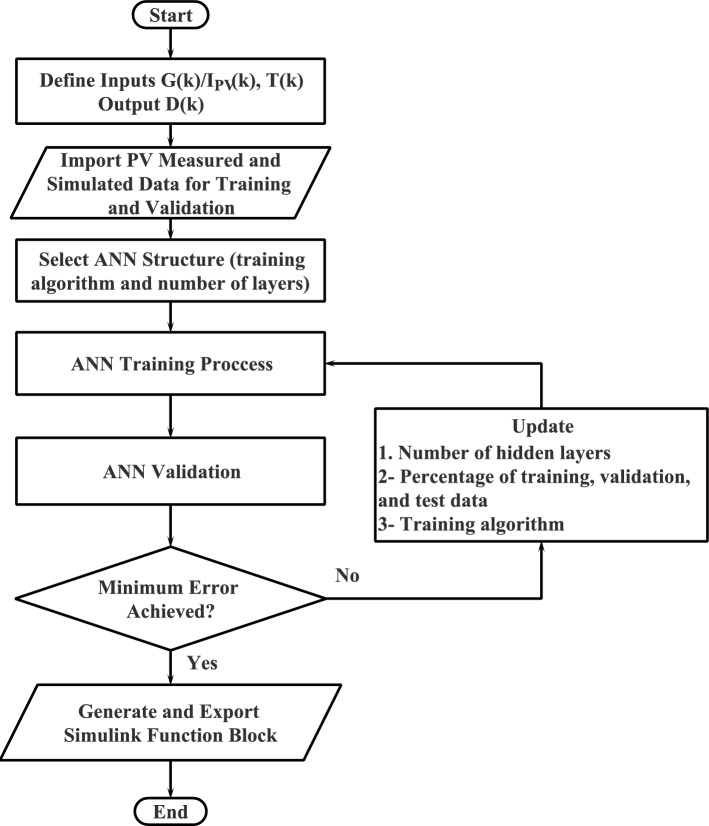
Flowchart of the training process for the proposed ANN-MPPT technique.

The suggested ANN-based MPPT method can be summarized according to the following stages:

1. **Data collection** Compile information on the power output of the solar panel and the relevant input variables, such as temperature, solar irradiance, and load.
2. **Data pre-processing** The data should be pre-processed to eliminate noise and disturbances as well as to standardize the data.
3. **Network training** Utilize the previously processed information to train the ANN using a suitable optimization strategy, such as backpropagation.
4. **Network validation** Use a different dataset to test the trained ANN’s accuracy and robustness.
5. **Implementation** Implement the trained ANN in the PV system to forecast the MPP and enhance the output power of the solar panels.

The flowchart of the training process is illustrated in Fig. 7, where *G*(*k*) denotes the present solar radiation, $I_{PV}(k)$ is the present PV panel current, *T*(*k*) is the present temperature of the panel, and *D*(*k*) denotes the present duty cycle.

#### Data collection

**Figure 8:**
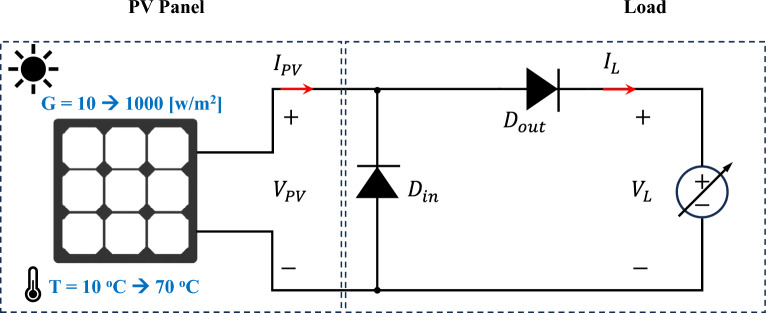
A schematic model for the data collection from the ReneSola-60W PV module.

**Figure 9:**
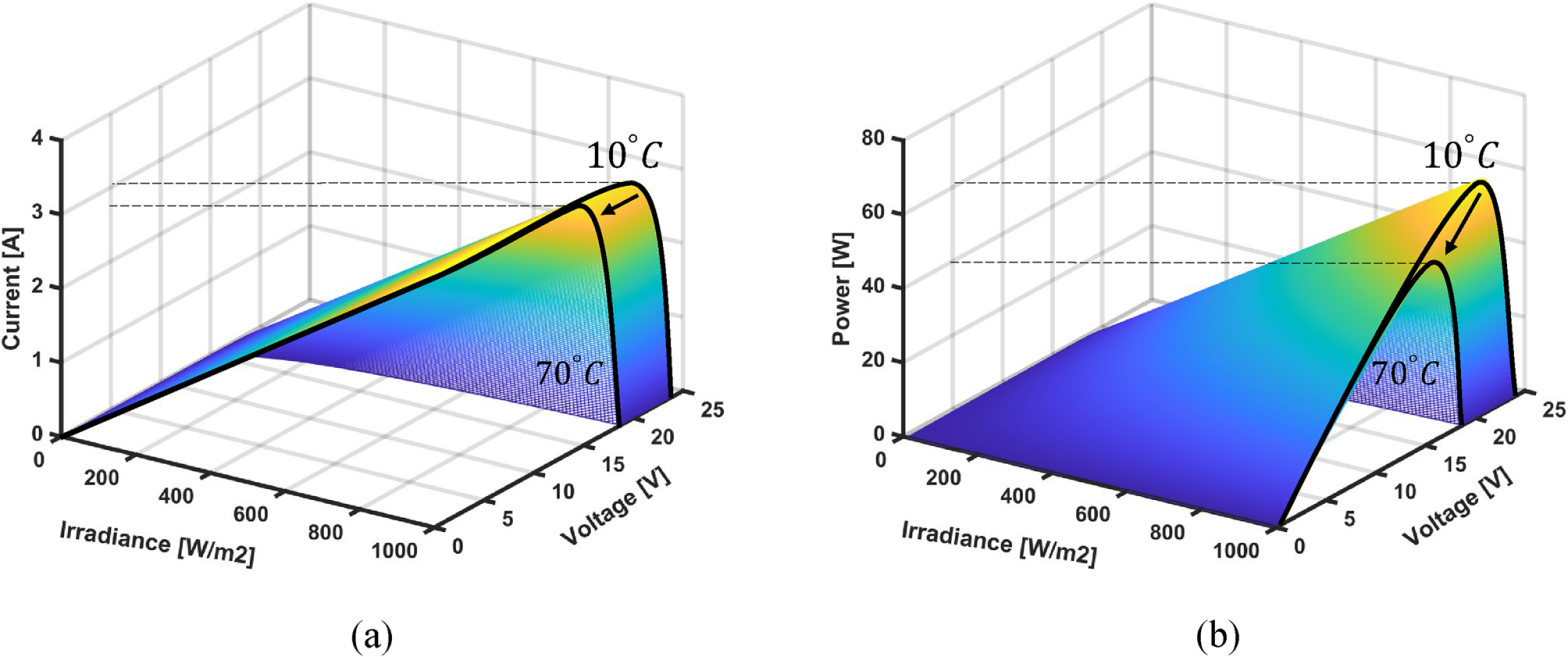
The characteristics of the ReneSola-60W PV panel under different radiation and temperature conditions: (**a**) I-V and (**b**) P-V characteristics.

**Figure 10:**
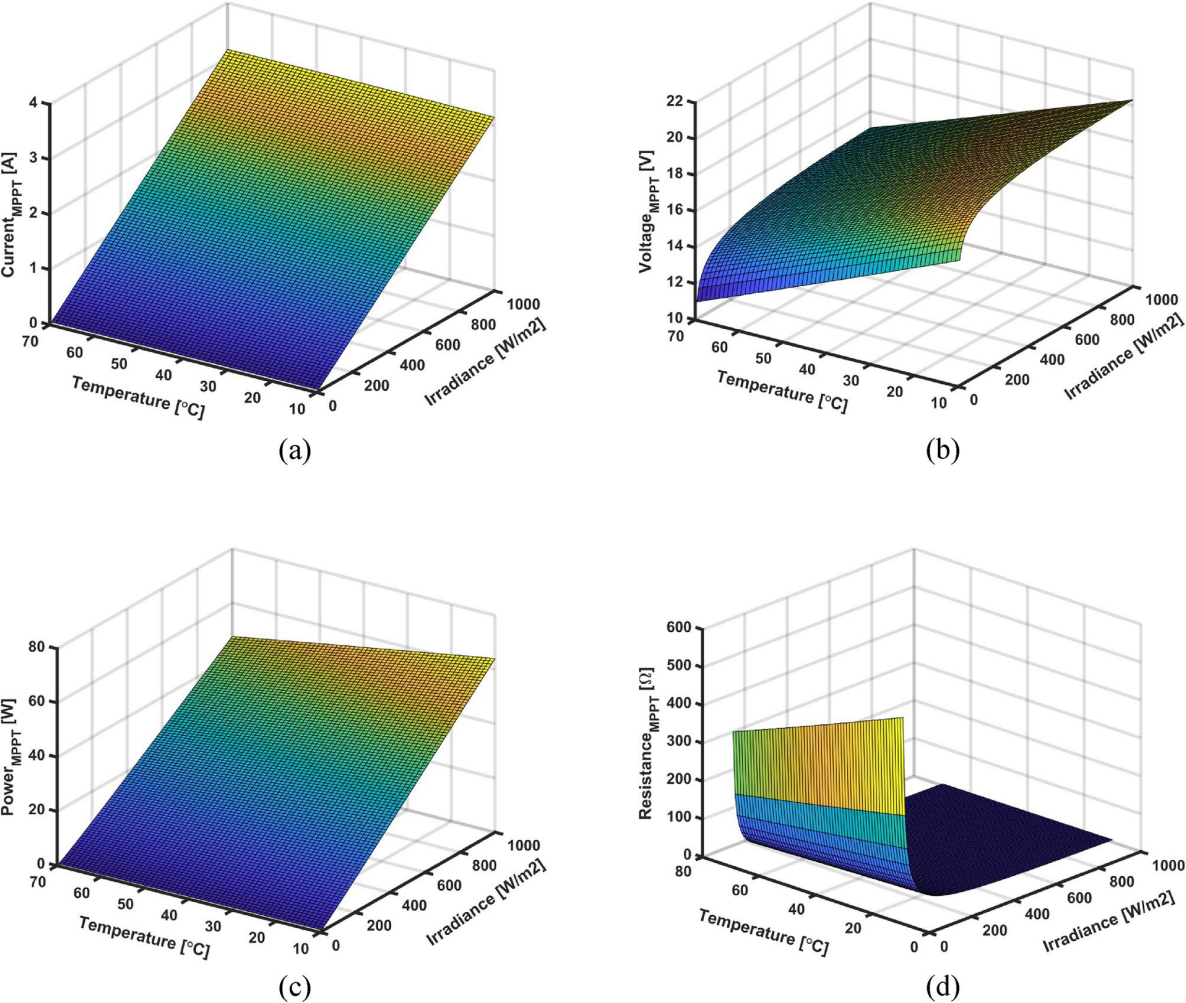
The ReneSola-60W PV module variables variation at MPP under different radiation and temperature conditions: (**a**) Current; (**b**) Voltage; (**c**) Power; and (**d**) Load resistance curves.

In this stage, numerous data have been collected from simulations and experiments where the measured noise has been filtered out. Then, the collected data is analyzed and stored in look-up tables to be used in the ANN training and validation stage. For this purpose, the target PV module has been connected to a variable DC source emulating the variation of the load resistance from open-circuit to short-circuit tests. A MATLAB/Simulink model has been developed for the ReneSola-60W PV module as seen in Fig. 8 where the irradiance is varied from 10 to 1000  W/m^2^ 70 °C. The specifications of the utilized PV panel are depicted in Table 2. The I–V and P–V characteristics of the ReneSola-60W PV panel for different temperatures and solar radiation conditions are shown in Fig. 9. The inverse relation between open-circuit voltage and temperature means that as the temperature rises, maximum power and open-circuit voltage decrease. Contrarily, the short-circuit current proportionally increases with solar radiation causing an increase in the maximum output power of the PV module. Similarly, the variation of the temperature has almost no effect on the current at MPP while the current remarkably increases with the radiation increase, as shown in Fig. 10a. The voltage at MPP is seen to be increasing by reducing the PV module temperature with a negligible increase in radiation, as depicted in Fig. 10b. Consequently, optimal power extraction can be attained at minimum temperature and maximum radiation on the PV module as shown in Fig. 10c. Additionally, it is found that the load resistance at the MPP follows an exponential decay behavior with radiation increase and a slight increase with temperature reduction, as demonstrated in Fig. 10d.

**Table 2:** Parameters of the employed PV panel ReneSola-60W.

Parameter	Value	Unit
$V_{OC}$	22.61	[V]
$I_{SC}$	3.38	[A]
$P_{mpp}$	60	[W]
$V_{mpp}$	19.01	[V]
$I_{mpp}$	3.16	[A]

#### Network training and validation

**Figure 11:**
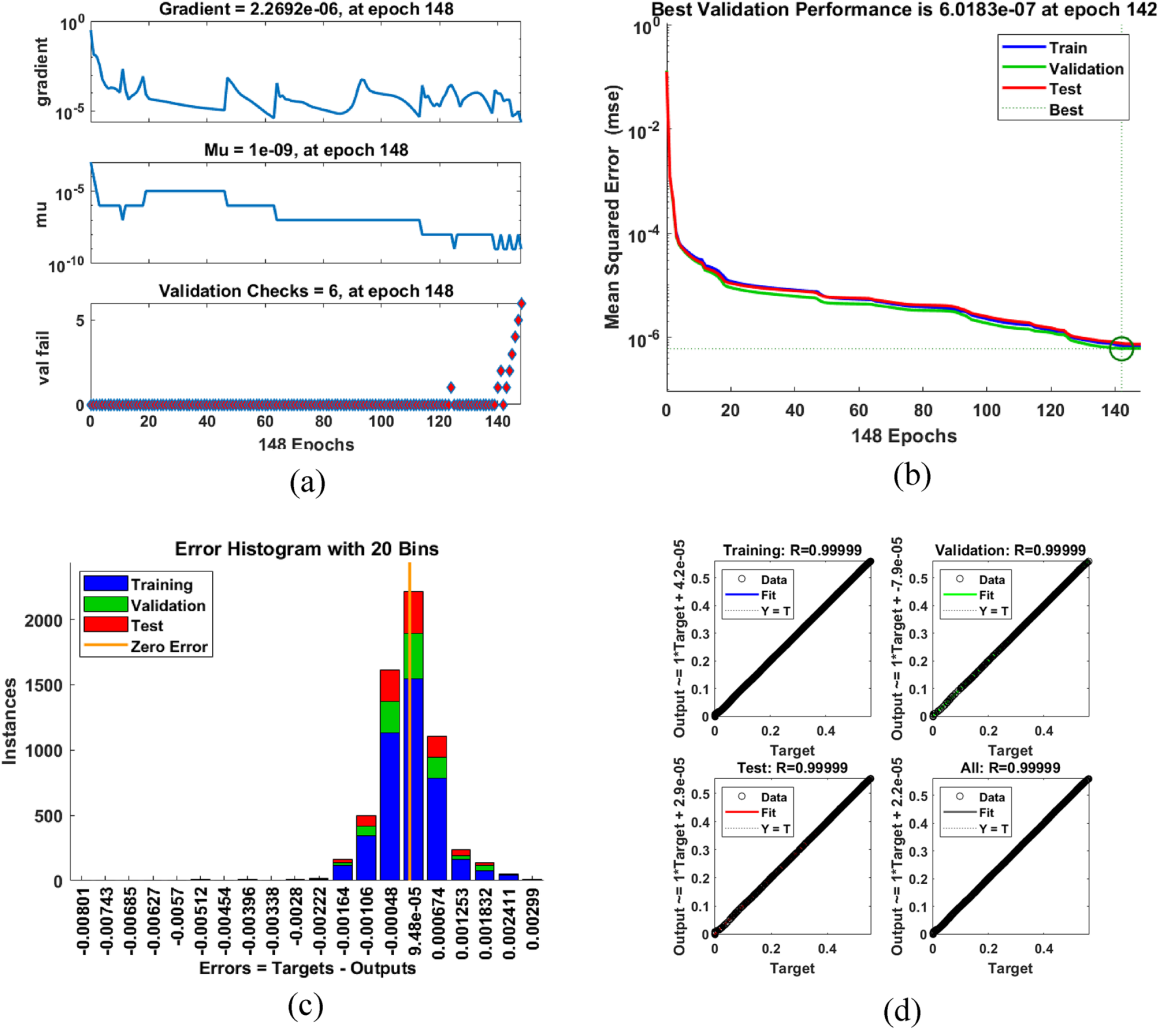
The proposed ANN-based MPPT technique training process and curve fitting tool considering radiation and temperature sensors, i.e., inputs (G,T) $\rightarrow$ output ($d_s$): (**a**) Training iterations; (**b**) Mean square error (MSE) during iterative process; (**c**) Error histogram and (**d**) Regression ratio.

**Figure 12:**
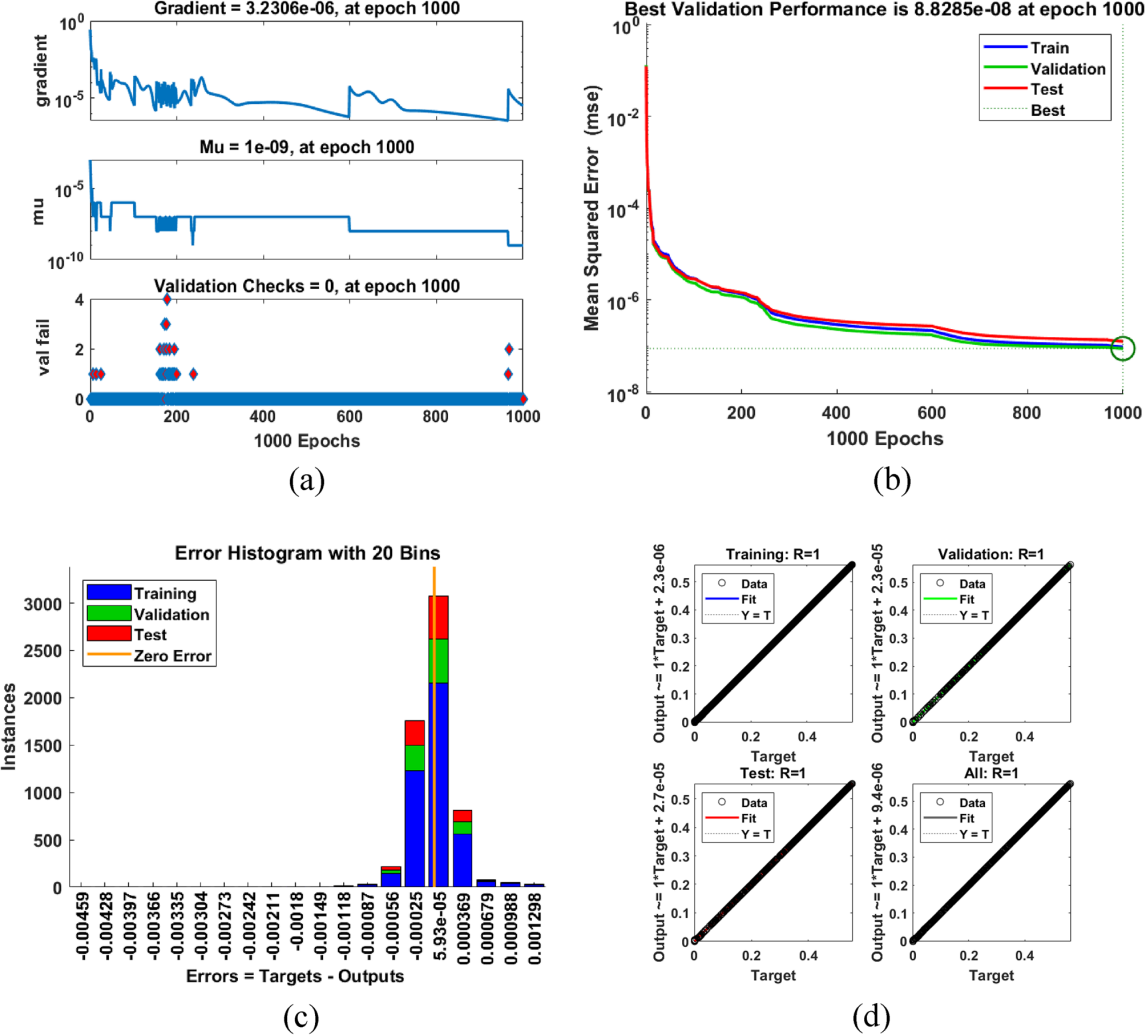
The proposed ANN-based MPPT technique training process and curve-fitting tool considering temperature and PV current sensors, i.e., inputs (T,I) $\rightarrow$ output ($d_s$): (**a**) Training iterations; (**b**) Mean square error (MSE) during iterative process; (**c**) Error histogram and (**d**) Regression ratio.

The input/output data stored from the previous stage is fed to the neural network MATLAB toolbox. Solar radiation, temperature, PV voltage, and/or PV current are used as the input signals of the ANN tool depending on sensor availability. On the other hand, the duty cycle of the switching device for the boost converter has been chosen as the output of the ANN function for implementation simplification. The input/output data which came from 6100 tests under different radiation and temperatures have been trained using Levenberg-Marquardt algorithm containing 10 layers with 70% of training data, 15% of test data, and 15% of validation data (see Fig. 11). The training process ended up with 148 epochs as seen in Fig. 11a while the Mean Square Error (MSE) reached its best validation performance at epoch 142 with 6.0183e−7 (see Fig. 11b). The error histogram in Fig. 11c shows that zero error between the target and the output has been achieved with a high regression factor (R = 0.99999) as seen in Fig. 11d. The same tests have been repeated considering current and temperature as the inputs and the duty cycle as the output of the ANN function (see Fig. 12). This alternative configuration is taken into consideration due to economic and implementation concerns.

#### Implementation of the proposed MPPT technique and optimal input sensors selection

**Figure 13:**
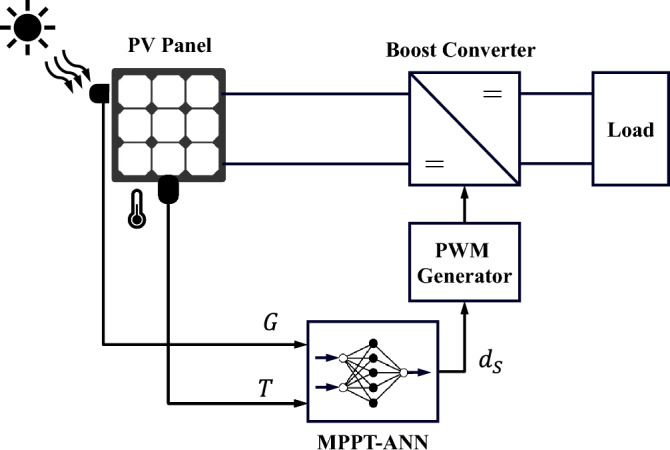
Schematic diagram of the traditional MPPT-ANN method using solar radiation and temperature sensors.

**Figure 14:**
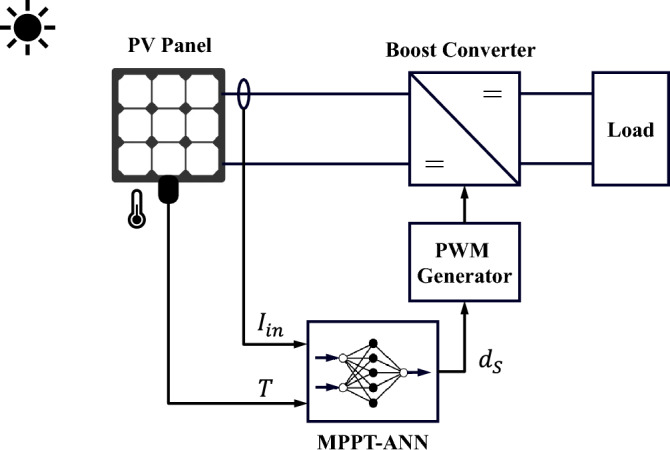
Schematic diagram of the proposed MPPT-ANN method using current and temperature sensors.

The final stage is to export the trained ANN model into Simulink for Simulation verification and hardware validation. The exported ANN fitting function is compatible with MATLAB Embedded Coder toolbox which can be implemented on common microcontrollers. A direct approach to implementing the suggested ANN-based MPPT is to measure the solar radiation and the temperature of the utilized PV panel (see Fig. 11). In this way, the ANN function correlates the input sensed signals into the trained data obtained from the I–V and P–V curves to the output duty cycle utilized for switching the boost converter through the Pulse-Width Modulator (PWM). Because of the high cost of the solar radiation sensor, the approach in Fig. 13, is not benefitable in small-scale photovoltaic applications such as battery charging systems or even to be installed in low-income rural areas. Therefore, an alternative approach is to replace the solar radiation sensor with a more affordable current sensor as shown in Fig. 14. This replacement is acceptable as the current increases proportionally with the light intensity subjected to the PV module. For temperature sensing, a conventional thermocouple can be used because of its cost-effectiveness, interchangeability, extensive measuring range, and reliability.

## Results and discussion

In this section, the selected MPPT methods discussed in section “Overview of existing MPPT techniques and the proposed ANN-MPPT technique in section “Proposed data-driven MPPT technique using artificial neural network” have been evaluated and compared by means of simulation using the MATLAB environment in section “Simulation results” . Then, the proposed ANN-MPPT is validated experimentally and compared to traditional MPPT techniques in section “Experimental validation”. The ReneSola-60W PV module characteristics under investigation, mentioned in section “Proposed data-driven MPPT technique using artificial neural network”, are provided in Table 2. The implemented PV system parameters including the converter and the load are provided in Table 3 where the switching frequency equals 10 kHz. MATLAB/Simulink is used to model the MPPT controllers based on the suggested techniques for various weather conditions which is an acceptable way of analyzing the performance of each above-mentioned technique.

**Table 3:** Parameters of boost converter and load.

Parameter	Value	Unit
$C_{in}$	100	[μF]
$C_{out}$	400	[μF]
$L_{c}$	1.5	[mH]
$R_{out}$	25	[$\Omega$]

### Simulation results

**Figure 15:**
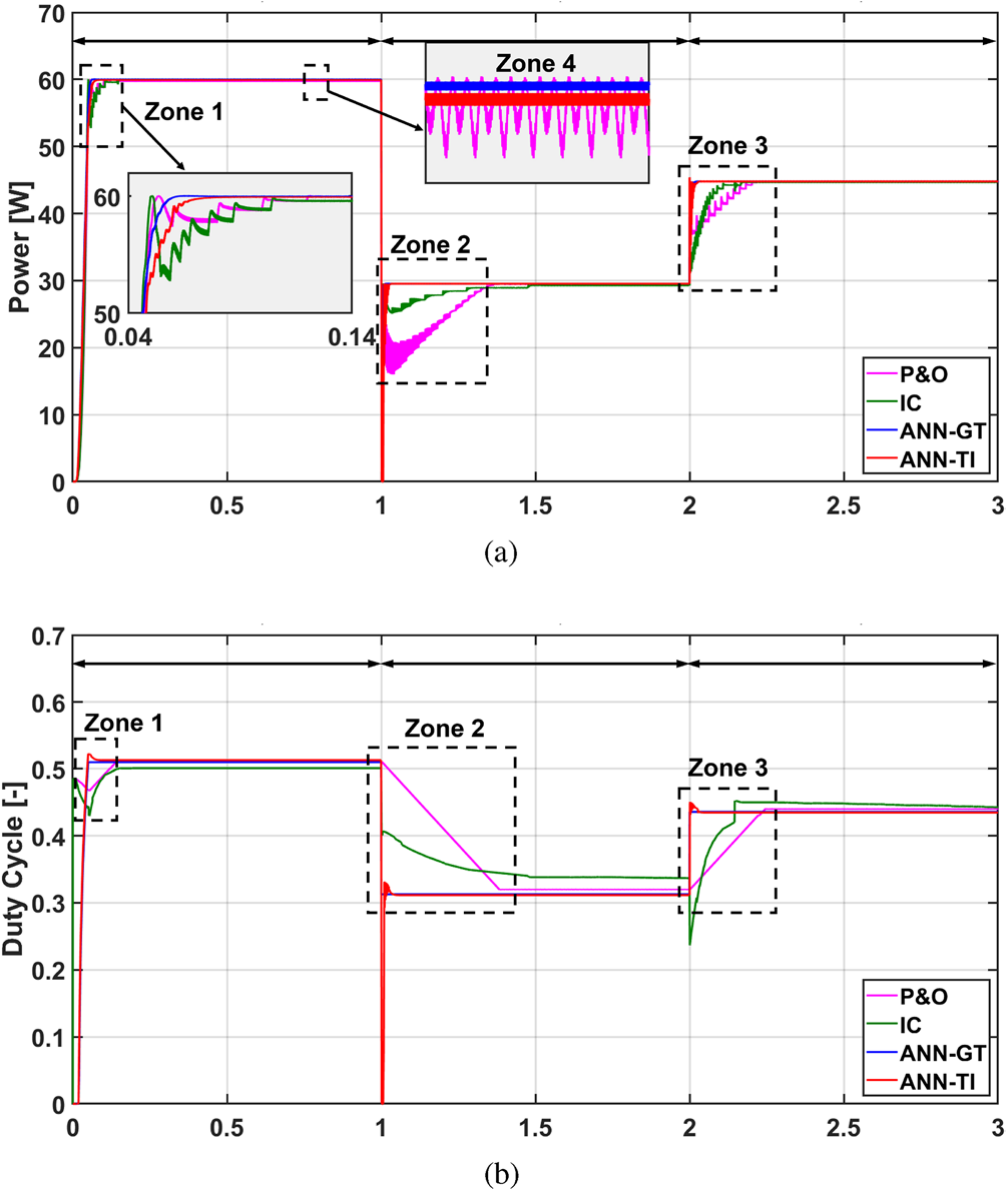
Simulation results of the presented MPPT methods under irradiance change (1000 $\rightarrow$500 $\rightarrow$750  W/m^2^) and constant temperature (25 °C): (**a**) Output power and (**b**) Duty cycle.

The demonstration and evaluation of the MPPT techniques under various weather conditions is a critical topic in PV systems. For the following simulation cases, the effectiveness and robustness of the suggested ANN-MPPT technique in optimizing the PV system’s energy extraction subjected to various weather scenarios will be addressed. In addition, a simulation comparison with conventional MPPT techniques like P&O and IC will be provided. The PV system conventional and proposed configurations presented in Fig. 1, 13, and 14 respectively are simulated under identical conditions. Three simulation scenarios are considered for the performance evaluation of the MPPT techniques P&O, IC, ANN-GT, and ANN-TI under the standard test conditions (STCs). In the first scenario, the radiation is set to 1000  W/m^2^ then decreased to 500  W/m^2^, and finally increased to 750  W/m^2^ at t = 0 s, t = 1 s, and t = 2 s, respectively. The temperature is kept constant at 25 °C during the whole test. From Fig. 15, it can be seen that ANN-based techniques (ANN-GT and ANN-TI) have almost identical behavior with the fastest convergence speed which reaches the MPP within 0.03 s at various irradiance levels. IC is seen to be following the ANN techniques with a convergence speed of 0.08 s at zone 1, 0.4 s at zone 2, and 0.2 s at zone 3 (see Fig. 15a). P&O shows the worst performance with the longest convergence speed of 0.08 s at zone 1, 0.3 s at zone 2, and 0.25 s at zone 3, besides the steady-state oscillatory behavior around the MPP seen in zone 4 Fig. 15a. The MPP achieved 60 W at 1000  W/m^2^, 29.5 W at 500  W/m^2^, and 45 W at 750  W/m^2^. The corresponding duty cycles of the simulated techniques are depicted in Fig. 15b.

**Figure 16:**
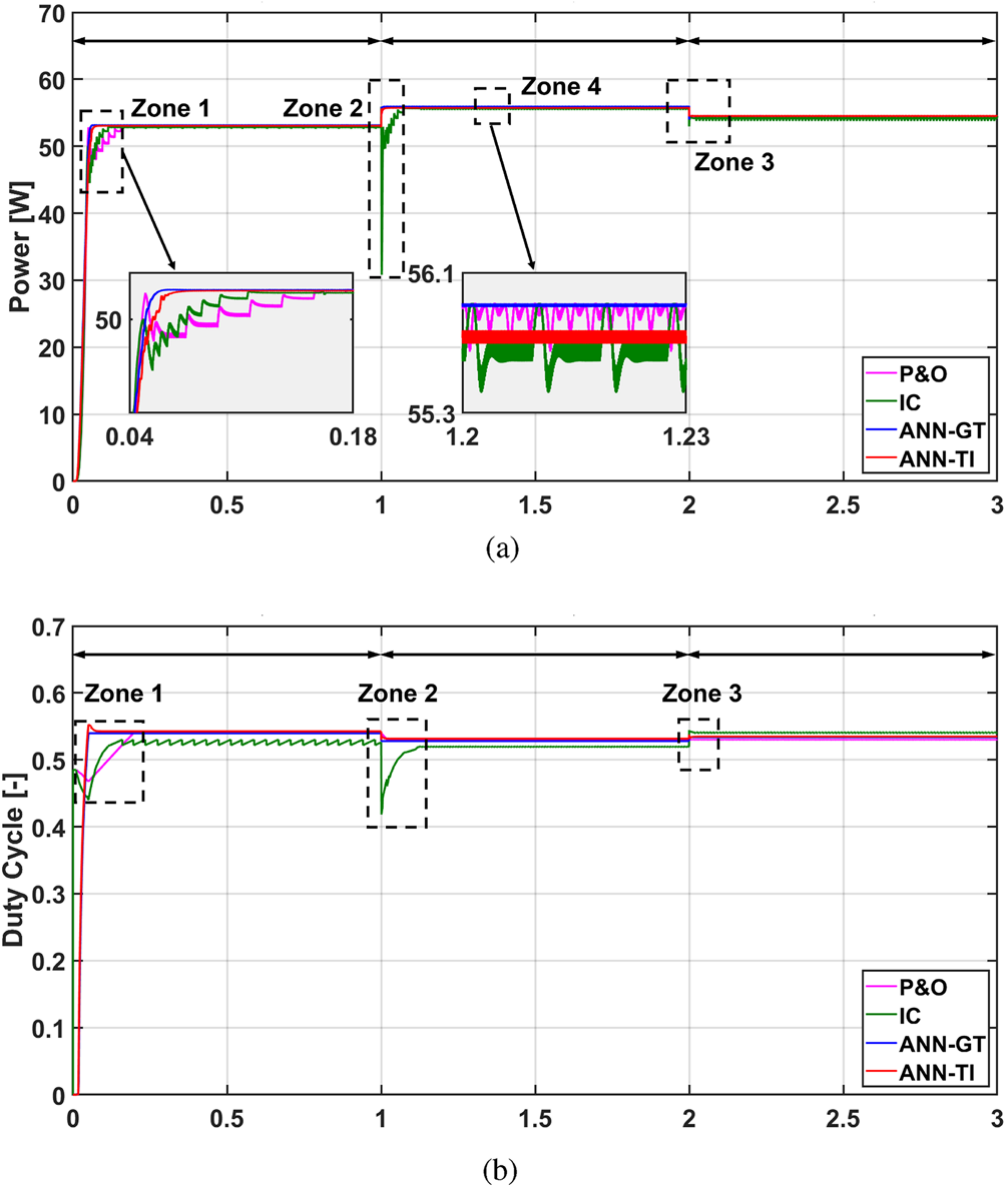
Simulation results of the presented MPPT methods under constant irradiance (1000 W/m^2^) and temperature change (50 °C $\rightarrow$ 40 °C $\rightarrow$ 45 °C): (**a**) Output power and (**b**) Duty cycle.

For the second scenario, the temperature is varied to 50 °C, 40 °C, and 45 °C at t = 0 s, t = 1 s, and t = 2 s respectively while the radiation is kept constant at G = 1000 W/m^2^. This assumption is valid for the evaluation of the dynamics and the performance of the MPPT controllers. As seen in Fig. 16, the temperature variation has a very small impact on the MPP of the PV module as addressed in section “Data collection” . However, the temperature variation showed a high impact on the IC tracking capability during the transients (see Zones 2 in Fig. 16a). The corresponding duty cycles of the simulated techniques under varying temperature and constant radiation are depicted in Fig. 16b. The proposed ANN-MPPT methods showed a robust performance with maximum convergence speed while P&O showed a moderate performance, as depicted in Fig. 16.

**Figure 17:**
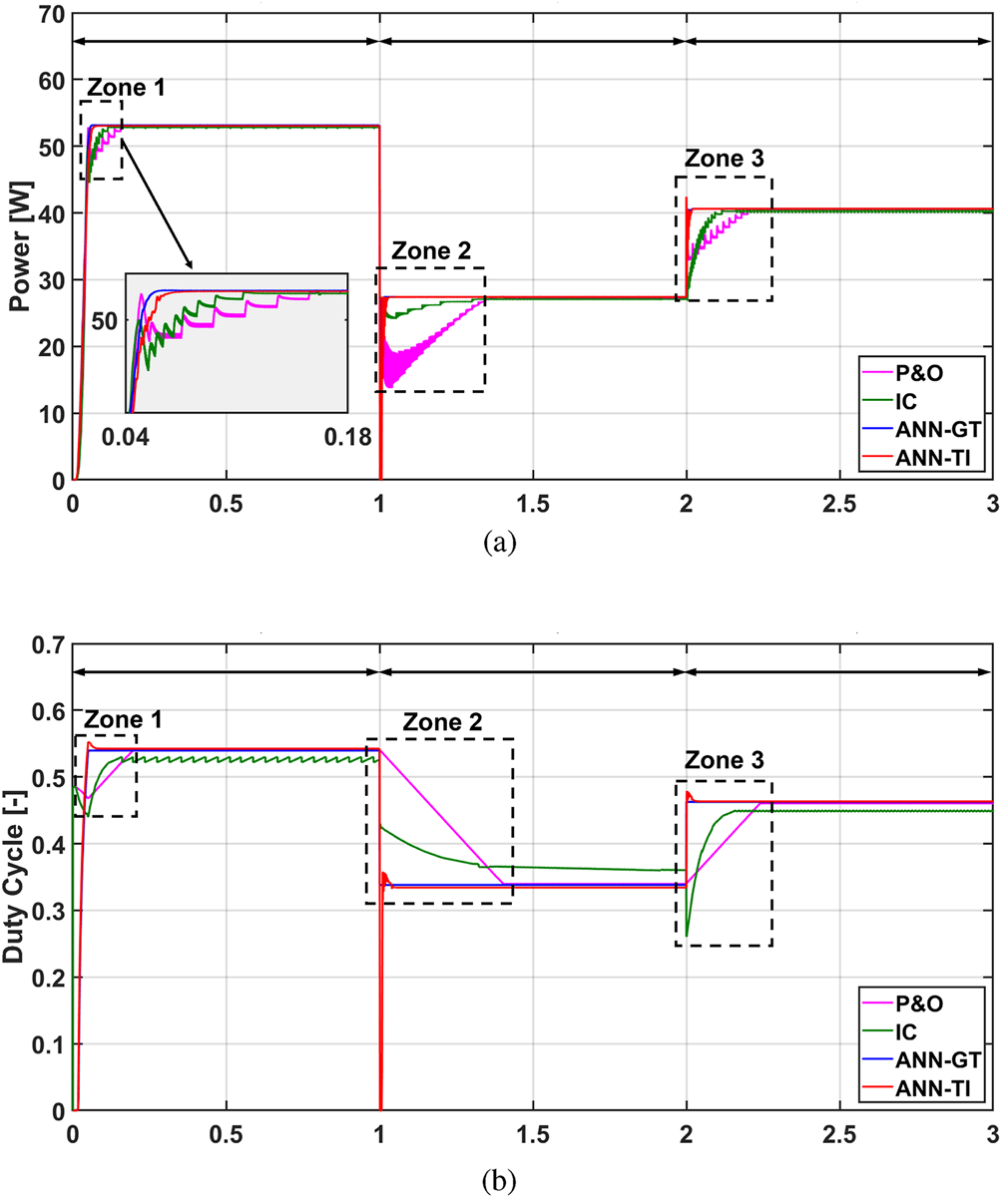
Simulation results of the presented MPPT methods under irradiance change (1000$\rightarrow$500 $\rightarrow$ 750 W/m^2^) and temperature change (50 °C $\rightarrow$ 40 °C $\rightarrow$ 45 °C): (**a**) Output power and (**b**) Duty cycle.

Finally, the combined effect of the radiation and temperature change is simulated in Fig. 17. It can be seen that the findings and conclusions of the transient period derived in this case are congruent with those of the first case. The steady-state MPP reduced to 53 W at 1000 W/m^2^, 27.4 W at 500 W/m^2^, and 40.6 W at 750 W/m^2^ (see Fig. 17a,b). Therefore, the reduction in the power generation at the MPP is due to the increase in the temperature as addressed in Fig. 10. However, the proposed ANN-GT and ANN-TI techniques show robust performance with higher MPP tracking capability compared to the IC and P&O MPPT methods.

### Experimental validation

**Figure 18:**
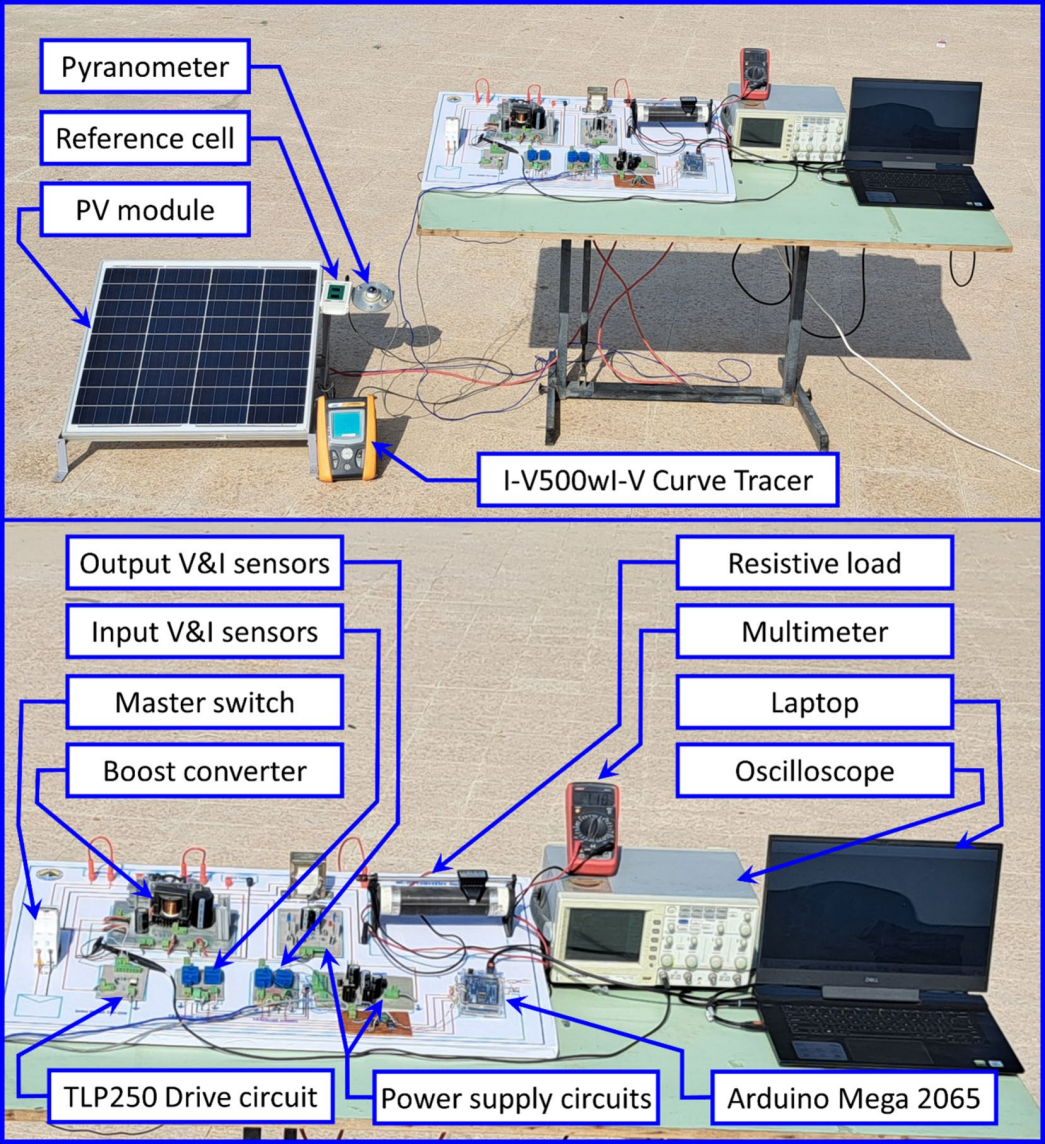
Experimental setup for the implementation of the MPPT techniques including the proposed ANN-based MPPT controller.

In this section, the efficacy of the MPPT controller based on the suggested ANN technique for an actual photovoltaic (PV) panel under varying radiation levels was experimentally assessed and validated. The experimental setup, illustrated in Fig. 18, includes two measuring circuits equipped with four LEM transducers (LV-25P and LA-25NP) to measure the PV module’s voltage and current, as well as the load for monitoring and control purposes. The specifications of the measurement devices are detailed in Table 4. The MPPT algorithms are implemented on an Arduino MEGA-2560, which generates PWM signals for the TLP250 opto-isolator drive circuit. The driver circuit is an intermediary between the control and power circuits, providing the triggering signals to the MOSFET. A switching frequency of 31.372 kHz is selected. The Arduino MEGA 2560 processor is used for deploying the ANN via the MATLAB support package for Arduino hardware. The deployment process involves the conversion of the trained ANN model into a transfer function compatible with the limited computational resources of the Arduino MEGA 2560. The well-implementation of the deployed function and the timely response of the controller, successfully tracking the MPP, evidences the performance of the ANN on the Arduino MEGA 2560. Although the Arduino MEGA 2560 is not as powerful as more advanced microcontrollers, simpler ANN tasks can still be effectively handled, making it suitable for basic MPPT and other low-cost, small-scale applications where real-time processing demands are not extremely high. The P–V characteristics of the ReneSola-60W PV module are obtained for different solar radiation using a solar analyzer of type (I–V500wI–V Curve Tracer). The variation in radiation has been done by utilizing transparent sheets that cover the surface of the ReneSola-60W PV module.

**Table 4:** Specifications of hardware elements and measurement devices.

Element		Description
PV module		ReneSola-60W
Boost converter	Switch	MOSFET (IRFP450)
	Switching frequency	$f_{sw}$ = 31.372 kHz
	Inductor	$L_c$ = 2.6 mH
	Output capacitor	$C_{out}$ = 1000 μF
	Input capacitor	$C_{in}$ = 330 μF
	Diode	D92-02N
LEM	Voltage sensor	LV-25P
	Current sensor	LA-25NP
Load		25 $\Omega$ Rheostat
Micro-controller		Arduino MEGA-2560
Solar analyzer		I–V 500 W I–V curve tracer
Radiation sensor		CMP21 pyranometer
Temperature sensor		Type-J thermocouple

Assuming that the measured variables are uniformly distributed, Type B standard uncertainty in (13) has been chosen for the experimental instrument’s uncertainty assessments where ‘*a*’ represents the instrument accuracy, and ‘*z* ’ signifies the standard uncertainty^59^. Table 5 provides details on the accuracy and standard uncertainty values for the measurement devices.

13 $$\begin{aligned} z&=\frac{a}{\sqrt{3}} \end{aligned}$$

The accuracy of all measured variables, including voltage, current, and solar irradiance, influences the ultimate uncertainty level of the calculated photovoltaic electrical efficiency ($\eta$). Therefore, the assessment of the calculated uncertainty in the electrical efficiency ($Y_\eta$), originating from uncertainties of all measured variables, was conducted utilizing the least-square fit method, as follows:

14 $$\begin{aligned} Y_\eta&=\left[ \left( \frac{\partial _\eta }{\partial _V}\right) ^2 \cdot z_V^2+\left( \frac{\partial _\eta }{\partial _I}\right) ^2\cdot z_I^2+\left( \frac{\partial _\eta }{\partial _G}\right) ^2\cdot z_G^2\right] ^\frac{1}{2} \end{aligned}$$

where $z_V$, $z_I$, and $z_G$ represent the precision of the measurement devices for voltage, current, and solar irradiance, respectively. Also, $\left( \frac{\partial _\eta }{\partial _V}\right)$, $\left( \frac{\partial _\eta }{\partial _I}\right)$, and $\left( \frac{\partial _\eta }{\partial _G}\right)$ represent the influence coefficients for voltage, current, and solar irradiance, respectively. By substituting the instrument uncertainties in Table 5 into equation (14), the obtained efficiency of the PV module is approximately 2.13% uncertain.

**Table 5:** Details of the experimental instruments.

Device	Range	Accuracy	Standard uncertainty
Ammeter (A)	0–10	± 0.01	5.78 × 10^−3^
Voltmeter (V)	0–200	± 0.01	5.78 × 10^−3^
Thermocouple (°C)	0–250	0.05	2.89 × 10^−2^
Pyranometer (W/m^2^)	0–2200	0.02	11.56 × 10^−3^

**Figure 19:**
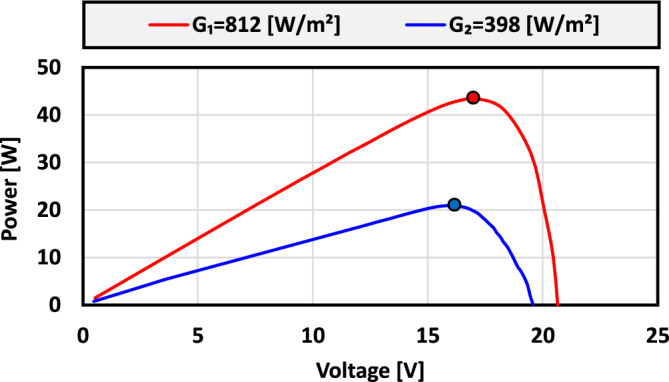
Experimental P–V characteristics of the ReneSola-60W PV module under variable radiation levels with constant temperature ($\approx 46 \; ^\circ \textrm{C}$).

At the beginning of the experimental tests, the solar analyzer shown in Fig. 18 was used to obtain the P–V characteristics of the RenoSola-60W module in both cases without and with transparent sheets (fully shaded PV module). It was found that the radiation during the test was 812 W/m^2^ with a maximum power of about 43.51 W while the radiation was reduced to 398 W/m^2^ by applying four transparent sheets achieved 20.94 W of MPP (see Fig. 19). The temperature measured during the experiments was found to be $\approx 46 \; ^\circ \textrm{C}$ with very small deviations $\pm 1 \; ^\circ \textrm{C}$. Therefore, the power change owing to temperature variation is negligible.

**Figure 20:**
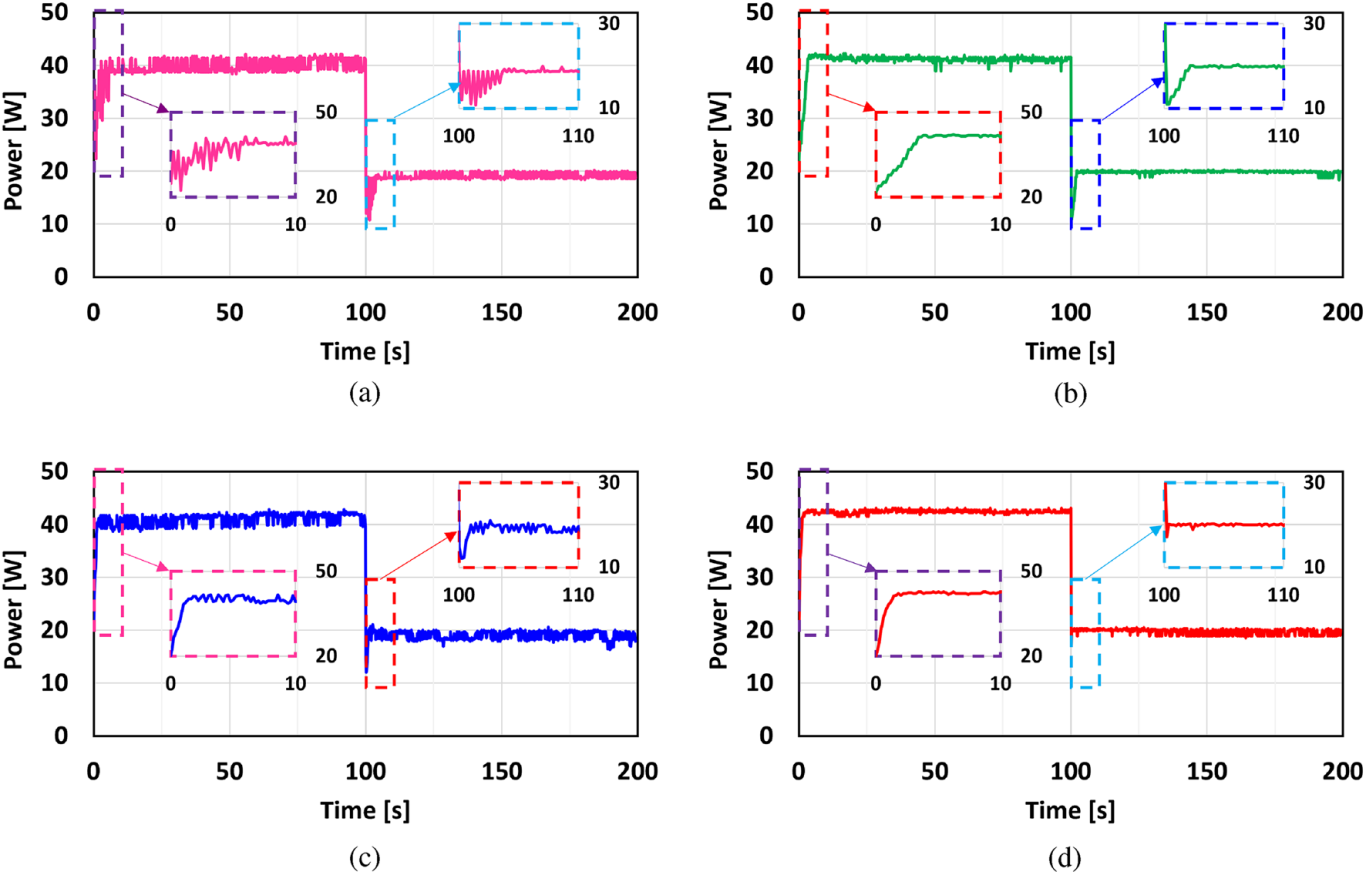
Experimental results of the presented MPPT methods under irradiance change (812 $\rightarrow$ 398 W/m^2^) and constant temperature ($\approx 46 \; ^\circ \textrm{C}$): (**a**) P&O; (**b**) IC; (**c**) ANN-GT; and (**d**) ANN-TI.

The experimental results of the aforementioned MPPT techniques: P&O, IC, ANN-GT, and ANN-TI are presented in Fig. 20a–d respectively. To fairly compare and evaluate all the MPPT techniques, all methods underwent identical conditions and test scenarios. Therefore, for all the following experiments, the PV module is subjected to natural radiation up to t = 100 s, and the four sheets are applied for another 100 s to emulate a fully shaded condition and reduce the received radiation by the PV module. Table 6 concludes the main findings of the experimental results for MPPT methods under investigation. It can be clearly observed that the ANN-TI showed the fastest tracking time amongst all methods while P&O is the slowest one. As expected, the results obtained are in agreement with theoretical analysis and simulation results for the P&O method, however, the ANN-TI shows a superior performance compared to ANN-GT regarding the tracking time and power fluctuations. This unexpected behavior is mainly due to the Pyranometer sensor nature which is used to feed the neural networks with radiation information. In contrast, the current sensor used in ANN-TI shows a faster response and less noise affected compared to the Pyranometer sensor. However, the ANN-GT method achieves the highest MPP tracking efficiency of 98.25% and 98.14% for 812 W/m^2^ and 398 W/m^2^ respectively. The IC method follows the ANN-GT from the overall performance perspective. The IC has a higher tracking time with lower tracking efficiency compared to other methods, however, it shows the minimum power fluctuations. This is due to the fact that low-pass filters are used in the input signals (voltage and current) instead of derivatives, as mentioned in section “Incremental conductance (IC)”, to find the MPP over the P–V curve. Thus, the low-pass filters should be designed carefully to compromise the dynamic response of the output filtered signals and the performance of the IC tracking algorithm.

**Table 6:** Experimental results comparison of the presented MPPT control techniques.

Operating condition	$G_1=812$ W/m^2^				$G_2=398$ W/m^2^			
MPPT controller	P&O	IC	ANN-GT	ANN-TI	P&O	IC	ANN-GT	ANN-TI
Output power [W]	42.06	42.06	42.75	42.71	20.18	20.29	20.55	20.52
Efficiency ($\eta _{MPPT}$ [%])	96.67	97.08	98.25	98.16	96.37	96.89	98.14	97.99
Tracking time [s]	6.4	3.6	1.6	1.3	3.8	2.4	1.4	0.4
Power fluctuations [%]	6.18	0.85	5.77	1.4	7.6	1.8	9.2	6.4

### Discussions

In this section, considering both simulation results and experimental validation, the overall performance of the MPPT techniques under consideration is elucidated. As expected, it is observed that the P&O technique showed the worst MPP tracking performance, characterized by elevated oscillations around the MPP during steady-state conditions. This leads to lengthy settling times and reduced efficiency in MPPT tracking. This is mainly owing to the tracking algorithm based on the perturbation of the duty cycle which requires a certain increase/decrease step every sample time regardless of the current state of the generated power. Additionally, it has been shown that the IC technique overcomes the weakness of the P&O method where IC showed a faster tracking performance with lower steady-state oscillations. The improved performance of the IC is derived from the I–V characteristics curve tracking through monitoring the current and voltage actual states and their derivatives. However, the main concern with the IC method is the measurements noise that deteriorate the tracking performance and could lead to failure of the tracking process and system instability. Thus, additional filtering is commonly imposed for proper operation of the IC technique at the cost of the MPP searching time. Finally, it has been illustrated that the suggested data-driven ANN-MPPT technique surpasses all other methods examined, exhibiting the shortest tracking time in both simulation and experimental outcomes. This technique achieves the highest MPPT efficiency along with the fastest tracking time. Additionally, its reliability and robustness are evident as it demonstrates the highest capability in tracking the MPP during rapid changes in temperature and solar radiation. Furthermore, the proposed technique is characterized by its simplicity in design, ease of implementation, and cost-effectiveness in terms of sensor requirements.

The stability of the ANN-MPPT method over extended periods and under varying conditions is recognized as crucial for assessing its reliability and longevity. The significance of investigating its performance under diverse weather scenarios is acknowledged to ensure its effectiveness in real-world applications. Further study on the long-term evaluation of the ANN-MPPT should be performed to provide a comprehensive understanding of the ANN-MPPT method’s stability and robustness.

## Conclusion

With the continuous evolution of technology, PV systems remain a cornerstone of renewable energy solutions which contribute to a more sustainable and eco-friendly energy landscape. MPPT plays a pivotal role in improving the performance of PV systems. Increasing energy efficiency, adapting the system’s dynamics according to variable weather conditions, and system stability are the main merits of involving the MPPT technique in PV systems. The MPP is the operating point where a PV system generates the highest energy level. As environmental conditions like solar irradiance, and temperature change frequently and in a non-linear manner, it becomes critical to monitor and respond to these changes to keep the PV’s operating point on the MPP. Due to their dependency on linear approximations, conventional approaches, such as P&O or IC, may be unable to model these non-linear connections effectively. Data-driven ANN algorithms provide an effective solution to this problem by exploiting their capacity to deal with non-linear correlations between input and output variables. This means that ANN may capture the complicated connections between environmental parameters and the related optimum operating point for MPP tracking. Additionally, by training on a wide set of inputs and outputs, ANN can be generalized effectively even with noisy or missing data enabling accurate MPP tracking under fast-changing conditions. Therefore, ANN overcomes the disadvantages of the conventional approaches, especially the IC method, which may fail because of measurement noise due to the presence of current and voltage signals derivates dI/dV. In this paper, a data-driven ANN-MPPT technique is suggested and compared to traditional MPPT techniques like P&O and IC. All methods have been simulated and implemented under identical conditions. The proposed technique showed a superior and robust performance with the highest MPP tracking capabilities compared to traditional methods under variable weather conditions. The proposed ANN-GT and ANN-TI achieve tracking efficiency of 98.25% and 98.16% respectively for 812 W/m^2^, 98.14% and 97.99% for 398 W/m^2^. The ANN-MPPT model was tested with diverse environmental conditions, showing effectiveness for selected PV panels. However, its generalization depends on training data diversity. Further validation with data from various locations and PV configurations is needed to confirm its general applicability. Future research should explore broader testing to enhance generalizability.

## Data Availability

The data that support the findings of this study are available from the author, A.R., upon reasonable request.
